# When technology meets aging: the paradoxical association between cognitive appraisal of AI and older employees’ successful aging at work

**DOI:** 10.3389/fpubh.2026.1956559

**Published:** 2026-10-07

**Authors:** Yujie Hao, Jiayi Wang

**Affiliations:** School of Public Administration, Nanfang College Guangzhou, Guangzhou, China

**Keywords:** cognitive appraisal of AI, job demands-resources model, older employees, SOC strategies, successful aging at work, work autonomy

## Abstract

**Objective:**

Against the dual backdrop of population aging and workplace digital transformation, promoting successful aging at work among older employees has become a critical issue in human resource management. Based on the Job Demands-Resources (JD-R) model, this study aims to examine the dual-pathway associations between older employees’ cognitive appraisal of artificial intelligence (AI) and their successful aging at work, as well as the mediating role of work autonomy and the moderating role of selective optimization with compensation (SOC) strategies.

**Methods:**

This study surveyed 558 employees aged 45 and above from various enterprises in China via questionnaires. Hierarchical regression analysis and Bootstrap approach were employed using Stata 17.0 to test the relationships among variables.

**Results:**

The findings indicated that: (1) Older employees’ cognitive appraisal of AI exhibits a double-edged correlational pattern regarding their successful aging at work. When older employees appraise AI as a challenge (theoretically corresponding to the resource-gain pathway), it is positively associated with their successful aging at work; when they appraise AI as a threat (theoretically corresponding to the demand-loss pathway), this association becomes negative. (2) Work autonomy was a significant mediator in both associational pathways. (3) SOC strategies positively moderated the relationship between challenge appraisal of AI (theoretically corresponding to the resource-gain pathway) and work autonomy, and buffered the negative relationship between threat appraisal of AI (theoretically corresponding to the demand-loss pathway) and work autonomy. (4) Moderated mediation analyses revealed that SOC strategies strengthened the indirect effect in the challenge appraisal (perceived resource-gain) pathway and weakened the indirect effect in the threat appraisal (perceived demand-loss) pathway.

**Conclusion:**

This study sheds light on the differentiated mechanisms through which older employees’ cognitive appraisal of AI relates to their successful aging at work, extending the application of the JD-R model to digital work contexts. The findings provide empirical evidence and practical insights for enterprises to promote older employees’ sustainable development in the AI era by enhancing work autonomy and stimulating proactive adaptation behaviors.

## 1 Introduction

At the current stage, human societal development is characterized by two inevitable trends. The deepening global population aging process has led to a series of issues, including a shrinking working-age population and increasing pressures on older adult care and childcare, which impose higher demands on social and organizational human resource management. To alleviate labor supply pressures, an increasing number of countries have implemented delayed retirement policies to extend employees’ working lives (1), implying that the number of older employees in enterprises will continue to rise in the future.

Of particular note, many older employees in the later stages of their careers may experience age-related declines in physiological functions such as hearing and vision, as well as cognitive functions including attention and memory (2–4). However, it is important to recognize that older employees are a heterogeneous group, and the timing and extent of such declines vary considerably across individuals. These changes may lead to physical exhaustion at work in certain individuals, affecting their capacity to perform high-intensity tasks and their problem-solving abilities (5). Additionally, some older employees are prone to psychological withdrawal, manifested as a “short-timer mentality” when approaching retirement age. This mentality is characterized by diminished growth motivation, reduced work engagement, and passive, lethargic psychological states, which may result in late-career disengagement behaviors (6–8). Moreover, older employees are often perceived as workers with lower potential, and such stereotypes impose greater limitations on their job-seeking and promotion opportunities in the later stages of their careers (9). These circumstances demand more from corporate human resource management, rendering a “one-size-fits-all” approach no longer appropriate. Instead, enterprises should seek new management approaches to provide work support for older employees, thereby motivating them to continue creating value for the organization (10).

Another major trend is the increasing integration of artificial intelligence into the workplace. While AI’s objective presence in organizations continues to grow, how employees subjectively appraise AI-related demands may be more consequential for their work outcomes. Existing research presents divergent views on how employees’ cognitive appraisal of AI relates to work attitudes and performance. From a positive perspective, when AI is appraised as a challenge, it has given rise to a new human-machine collaborative work model (11), which can not only replace routine, repetitive tasks (12) but also enhance employees’ personal work capabilities and prompt them to proactively update their technical skills (13). From a negative perspective, the new job competency requirements brought by AI may increase employee work stress and lead to AI-related fears (14). For older employees, who are already at risk of declining work quality and marginalization in their later careers, subjectively appraising AI as a threat may further exacerbate their job insecurity (15–17). Due to age-related changes in cognitive functions that vary across individuals, some older employees may face greater practical constraints in the digital workplace (18, 19).

While resource depletion due to aging can negatively impact older employees, aging is a natural and largely irreversible process, and workplace aging is an important issue that both enterprises and employees must ultimately confront. Against the backdrop of deepening population aging and workplace digitalization, it is urgent to help older employees adapt to the new work demands brought by AI. This involves fully tapping and unleashing their work potential, and ultimately achieving successful aging at work. Addressing this challenge is both an urgent necessity to alleviate current social labor supply pressures and a practical issue that enterprises and society must continuously address in the future.

Successful aging at work refers to older employees’ ability to proactively maintain their capabilities and levels commensurate with work demands to sustain working status (20). It is a dynamic process requiring continuous self-regulation by older employees to ultimately achieve person-environment fit (21), representing a positive perspective on employee aging. Older employees who achieve successful aging at work possess strong subjective initiative, problem-solving abilities, work motivation, and retention willingness, enabling them to continuously create value for the organization and serve as vital human capital for enterprises (22, 23).

Successful aging at work results from the joint efforts of enterprises and older employees. Current scholars frequently employ the Job Demands-Resources (JD-R) model to explore the mechanisms through which working conditions affect successful aging at work. The JD-R model categorizes various workplace factors into job demands and job resources. Job demands refer to the sustained physical and mental efforts employees must expend to meet work requirements, entailing certain physiological and psychological costs. Job resources refer to various physiological, psychological, and social resources that help employees achieve work goals, reduce job demands, and promote personal growth (24). How older employees appraise AI in the workplace can be theoretically linked to both demand consumption and resource empowerment. When appraised as a threat, AI is often perceived as a technostressor, potentially leading to poor employee performance. When AI is appraised as a challenge, it can provide effective assistance through cognitive compensation and autonomous learning support, which may facilitate personal growth and capability enhancement. Thus, employees’ cognitive appraisal of AI may show a double-edged associational pattern with their work outcomes.

Furthermore, work autonomy is considered the most frequently utilized resource for buffering the effects of job demands (25). Work autonomy refers to employees’ discretion in their work (26). Granting older employees a certain degree of work autonomy can effectively motivate their work capabilities and motivation (27, 28). When older employees have higher work autonomy, they are more likely to invest more time and energy in learning new technologies to improve task efficiency, and maintain a learning state to sustain capabilities commensurate with work demands, thereby facilitating successful aging at work.

In addition to organizational work support, older employees also need to adopt proactive adaptation strategies to maintain and acquire necessary work resources. Among these, selective optimization with compensation (SOC) strategies are the most widely applied. SOC strategies, as dynamic adaptation strategies, refer to individuals’ use of selection, optimization, and compensation as three coordinated mechanisms to set and achieve goals when facing resource decline challenges (29). Employees who effectively utilize SOC strategies allocate and utilize resources more efficiently. These strategies effectively mitigate age-related functional decline (30), help older employees strengthen goal selection during work, and continuously optimize work processes through proactive learning (31, 32). Therefore, this study posits that SOC strategies constitute an important job resource and a critical condition for achieving successful aging at work.

China has currently entered a moderately aging society, with the degree of aging continuing to deepen. To address a series of issues brought by population aging (33), China has begun implementing delayed retirement policies to institutionally extend employees’ labor participation cycles. Workplace aging is becoming an irreversible structural feature of Chinese society and organizational development. Due to cultural differences between China and the West, research on successful aging at work in the Chinese context must proceed from China’s specific realities. Chinese culture advocates collectivism, emphasizing self-concept construction defined in relation to social context and the relationship between humans and nature (34). Thus, research on successful aging at work should attend to employees’ personal workplace experiences, focusing on the positive emotions and growth perceptions they gain in their later career stages (35).

Although prior research has examined how AI relates to employee attitudes and performance, several gaps remain. Most studies focus on AI adoption, objective exposure, or technology acceptance among general or younger employees, whereas older employees’ successful aging at work has received limited attention. Existing findings on AI’s effects are also mixed, and few studies adopt a cognitive appraisal perspective to distinguish challenge and threat appraisals as theoretically distinct resource-gain and demand-loss pathways. Moreover, the mediating role of work autonomy and the moderating role of SOC strategies have rarely been tested in an integrative model, especially in the Chinese context of population aging and delayed retirement. To address these gaps, this study draws on the JD-R model and examines three questions: (1) whether challenge and threat appraisals of AI are differentially associated with older employees’ successful aging at work; (2) whether work autonomy mediates these associations; and (3) whether SOC strategies moderate these mediating pathways. Grounded in China’s specific national conditions, this research holds significant practical implications for Chinese enterprises in motivating and developing older human resources and optimizing human resource management practices.

## 2 Theoretical basis

### 2.1 The relationship between older employees’ cognitive appraisal of AI and successful aging at work

The role of older employees’ cognitive appraisal of AI in their successful aging at work essentially concerns how such appraisal relates to employees’ personal resource balance—that is, whether AI is appraised as a job resource helping employees supplement and enhance personal resources, or as a job demand accelerating resource depletion. The academic community has yet to reach a consensus on this issue. Studies supporting positive effects argue that when AI is appraised as a challenge (theoretically corresponding to a job resource), its application in task automation and technological empowerment can replace older employees’ routine, repetitive work to reduce their burden, while also alleviating difficulties arising from age-related physical changes that vary across individuals in performing high-intensity tasks (36). Although some older employees may face considerable limitations in adapting to new work demands due to physiological and cognitive resource depletion and weak emerging skills (18, 19, 37), when AI is appraised as a challenge, AI technology itself can help compensate for their age-related skills change and facilitate intergenerational knowledge transfer (38–40). When older employees can recognize and proactively utilize this resource, they can achieve career growth, and their willingness to extend working lives increases (41). Studies supporting negative effects generally regard AI, when appraised as a threat (theoretically corresponding to a job demand), as a technostressor (42), because its application may replace existing positions, thereby increasing work pressure and anxiety (17). The human-machine collaboration proficiency required by AI application forces older employees into a “re-learning cycle,” and the learning fatigue from this cycle depletes their cognitive and emotional resources (43). The reshaping of work methods by emerging technologies may plunge some older employees into role ambiguity and identity threat, accelerating psychological resource depletion (39, 44).

How employees cognitively appraise AI plays a crucial role in shaping its outcomes. According to Lei and Ngai’s (45) cognitive technostress appraisal framework, individuals can appraise technology-related demands either as a challenge or as a threat. A challenge appraisal is theorized to correspond to the resource-gain pathway, whereas a threat appraisal is theorized to correspond to the demand-loss pathway. These are theoretical correspondences, not direct measurement equivalences. These two appraisal patterns are theorized to have divergent effects on older employees’ successful aging at work. Based on the above analysis, this study proposes the following hypotheses:

*H1*: Older employees’ cognitive appraisal of AI is associated with their successful aging at work.

*H1a*: When AI is appraised as a challenge (theoretically corresponding to AI as a job resource), it is positively associated with older employees’ successful aging at work.

*H1b*: When AI is appraised as a threat (theoretically corresponding to AI as a job demand), it is negatively associated with older employees’ successful aging at work.

### 2.2 The mediating role of work autonomy

With increasing age, older employees need to maintain motivation by completing challenging and fulfilling work (46). Work autonomy, as the most common job resource, has been demonstrated to positively affect employees’ work attitudes and motivation (47, 48). According to Self-Determination Theory (SDT), autonomy is an indispensable resource for promoting personal growth and well-being. When the work environment satisfies autonomy needs, individuals exhibit stronger intrinsic motivation and higher well-being; conversely, frustration of autonomy needs leads to decreased motivation (49).

In the positive empowerment pathway, when AI is appraised as a challenge, it may help older employees handle complex tasks, make information transmission and recording more organized, enhance work schedule flexibility, and increase employees’ work autonomy (38). Granting work autonomy to employees can increase their sense of control and work engagement, promoting performance and stimulating creative willingness (50, 51). Older employees with higher autonomy are more likely to maintain a continuous learning state, while the information support function provided by AI offers them richer training resources (52, 53), which may facilitate their successful aging process. In the negative depletion pathway, however, when AI is appraised as a threat, algorithmic opacity may deplete employees’ decision-making capabilities and lead them to execute low-quality decisions, suppressing their autonomy needs (54). Algorithmic monitoring and evaluation can cause employees to lose their sense of work control (55), hindering intrinsic motivation and self-regulated learning, ultimately affecting successful aging at work.

As discussed above, the two appraisal patterns—challenge and threat—are theorized to correspond to the resource-gain and demand-loss pathways, respectively. We further propose that work autonomy mediates these relationships. Based on the above analysis, this study proposes the following hypotheses:

*H2*: Work autonomy mediates the relationship between cognitive appraisal of AI and older employees’ successful aging at work.

*H2a*: When AI is appraised as a challenge (reflecting AI as a job resource), work autonomy plays a positive mediating role.

*H2b*: When AI is appraised as a threat (reflecting AI as a job demand), work autonomy plays a negative mediating role.

### 2.3 The moderating role of SOC strategies

SOC strategies constitute a key theoretical framework for achieving loss minimization and gain maximization in the successful aging process, serving as a meta-model for studying successful aging (56). SOC strategies refer to individuals’ use of selection, optimization, and compensation strategies to cope with resource loss and maintain dynamic balance between resource loss and replenishment when facing limited physiological, psychological, and social resources (57). With increasing age, older employees’ physiological and social resources at work gradually deplete. This resource loss is a source of work stress (58). Beyond resource loss, employees must also cope with the complexity and uncertainty of the work environment in the AI era. Transactional Theory of Stress and Coping (TTSC) provides an important perspective for understanding individuals’ differentiated responses to environmental challenges. When older employees appraise AI as a challenge, they activate coping strategies promoting personal adaptation and growth; when AI is appraised as a hindrance, it triggers withdrawal and anxiety (59, 60). According to the JD-R model, SOC strategies, as important personal resources, play critical moderating roles in both pathways-amplifying the positive effects of job resources while buffering the negative impacts of job demands.

Employees’ proactive adjustment behaviors effectively maintain their work autonomy (61). The use of SOC strategies can enhance older employees’ acceptance of new technologies and promote successful aging through intelligent technology use (62, 63). When AI is appraised as a challenge (theoretically corresponding to a job resource), older employees employing SOC strategies can set, adjust, and pursue goals through optimizing resource allocation to ensure smooth task completion. Through AI technology, they proactively learn new skills and refine work methods to maintain and enhance their sense of work control (64, 65). This process helps them adapt to age-related personal resource changes and increasingly difficult work demands (62), which may improve job satisfaction and work autonomy. Such proactive behaviors in AI workplace scenarios can strengthen their work autonomy, facilitating the realization of successful aging at work by achieving coordination between personal capabilities and the organization in digital work contexts (40, 66).

Conversely, when AI is appraised as a threat (theoretically corresponding to a job demand) and creates pressure for older employees, those with high SOC strategies can maintain personal goals, combining social needs, personal capabilities, and intrinsic motivation to focus limited energy and resources on core objectives. They possess skills for autonomous goal selection and revision, better enabling them to select among the diverse work information provided by AI technology and develop new domain goals (30, 57). Simultaneously, they reasonably allocate time, energy, and knowledge to systematically enhance skills to achieve goals, buffering the negative impact of AI through the weakening of autonomy.

Given that challenge appraisal is theorized to correspond to a resource-gain pathway and threat appraisal is theorized to correspond to a demand-loss pathway, we further propose that SOC strategies moderate the relationships in both pathways. Based on the above analysis, this study proposes the following hypotheses:

*H3*: SOC strategies moderate the relationship between older employees’ cognitive appraisal of AI and older employees’ work autonomy.

*H3a*: When AI is appraised as a challenge (theoretically corresponding to AI as a job resource), SOC strategies positively moderate the relationship between challenge appraisal of AI and work autonomy.

*H3b*: When AI is appraised as a threat (theoretically corresponding to AI as a job demand), SOC strategies negatively moderate (i.e., buffer) the relationship between threat appraisal of AI and work autonomy.

### 2.4 Moderated mediation effects

Integrating the above arguments, this study proposes that SOC strategies not only moderate the relationship between older employees’ cognitive appraisal of AI and work autonomy, but also moderate the entire mediation process. The indirect association of older employees’ cognitive appraisal of AI with successful aging at work through work autonomy depends on the level of SOC strategies employed by older employees.

In the resource-gain pathway, when older employees can utilize SOC strategies to increase their acceptance of AI (63) and adopt proactive adaptation behaviors, the positive association of challenge appraisal of AI with work autonomy and its positive association with successful aging at work are both strengthened (39). SOC strategies serve as a necessary channel for translating personal capabilities into successful aging outcomes (32). In the demand-loss pathway, older employees who effectively employ SOC strategies can successfully cope with resource depletion, thereby weakening the negative association between threat appraisal of AI and autonomy (30, 32).

Based on the above analysis, this study proposes the following hypotheses:

*H4*: SOC strategies moderate the mediating effect of work autonomy in the relationship between older employees’ cognitive appraisal of AI and older employees’ successful aging at work.

*H4a*: When AI is appraised as a challenge (theoretically corresponding to AI as a job resource), SOC strategies positively moderate the mediating effect.

*H4b*: When AI is appraised as a threat (theoretically corresponding to AI as a job demand), SOC strategies negatively moderate (i.e., buffer) the mediating effect.

The research model of this study is shown in Figure 1.

**Figure 1:**
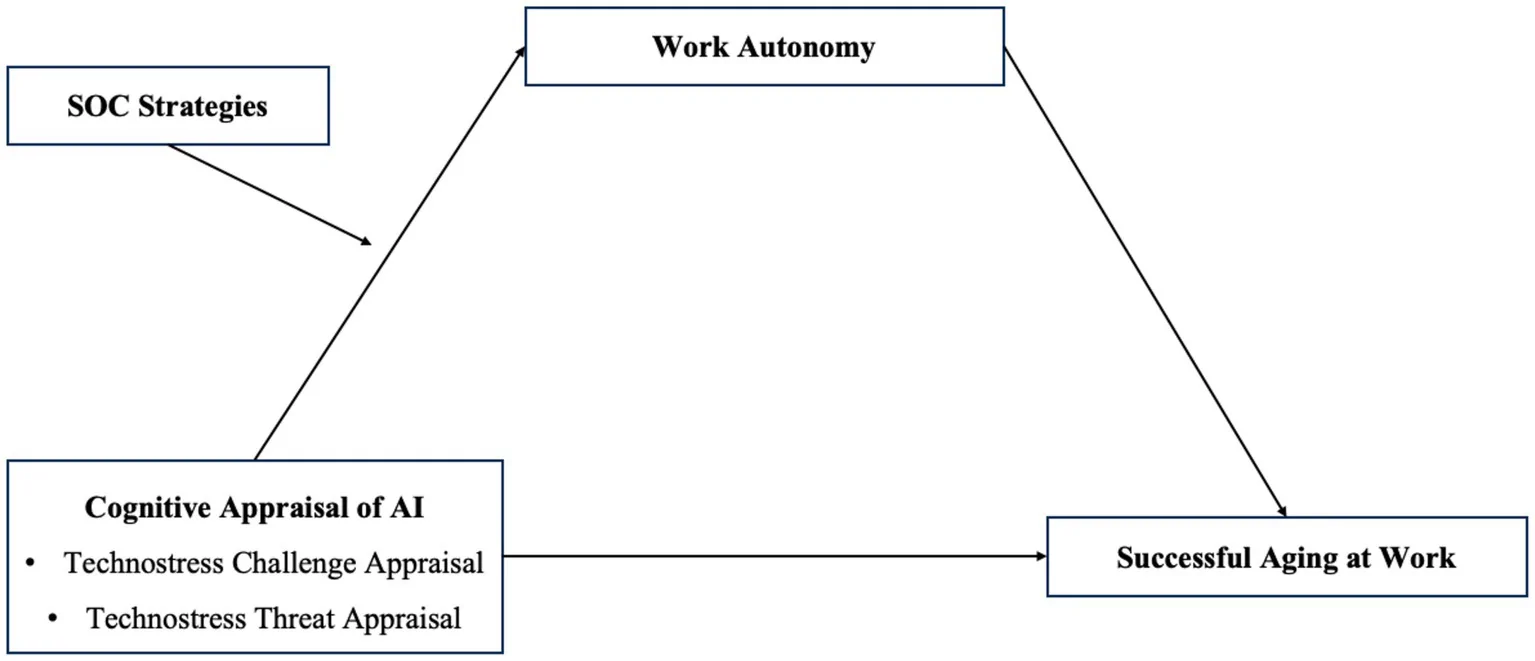
The moderated mediation model.

## 3 Research design

### 3.1 Data source

Previous studies have shown that employees experience natural physiological and cognitive decline after age 45, and their perception of remaining lifespan also changes significantly (40, 67). Therefore, this study focused on employees aged 45 and above working in various enterprises. Age was collected as a categorical variable with two groups: 45–55 years and 56 years and above.

Data were collected between September and December 2025 through online questionnaires and on-site surveys. Participants were employees aged 45 and above from cooperating enterprises in four cities in Guangdong Province, China: Guangzhou, Shenzhen, Foshan, and Dongguan. Partner enterprises were recruited through the research team’s institutional and enterprise contacts, and all eligible employees in these enterprises were invited to participate. The survey did not impose industry restrictions, and industry category was not recorded as a variable; firm ownership type is reported in Table 1.

**Table 1:** Demographic and occupational characteristics of the sample (N = 558).

Variable	Classification	Number	Percentage (%)
Gender	Male	291	52.15
	Female	267	47.85
Age	45–55 years	363	65.05
	56 years and above	195	34.95
Education	High school or below	268	48.03
	Higher Education (College)	234	41.94
	Higher Education (Bachelor+)	56	10.04
Position	Ordinary employee	327	58.6
	Junior manager	111	19.89
	Middle manager	81	14.52
	Senior manager	39	6.99
Firm type	State-owned enterprise	183	32.80
	Private enterprise	168	30.11
	Joint venture	63	11.29
	Foreign-owned enterprise	75	13.44
	Others	69	12.36

A total of 596 questionnaires were returned. After data cleaning, 558 valid responses were retained, yielding a valid response rate of 93.62%. Data cleaning followed three criteria: (1) responses with identical answers across all items (straight-lining) were removed; (2) responses failing attention-check items were excluded; (3) responses with missing values on key variables were deleted. Details of the sample characteristics are presented in Table 1.

To ensure that the appraisal scores reflected a real rather than hypothetical work context, all partner enterprises had introduced AI-related work systems (e.g., intelligent office assistants, algorithmic scheduling, or AI-supported customer-service tools) before data collection. A screening item at the beginning of the questionnaire confirmed that participants had directly used such AI-related systems in their daily work; employees without prior work experience with AI were instructed not to complete the survey.

### 3.2 Variable selection

The independent variable was older employees’ cognitive appraisal of AI in their enterprises. This study adopted the Cognitive Technostress Appraisal Scale developed by Lei and Ngai (45). The original scale was developed to measure general technology-related demands. To adapt it to the AI context, we replaced “information technology” with “artificial intelligence (AI)” in all items. For example, the original item “The demands arising from using information technology at work help me understand myself better” was reworded as “The demands arising from using AI at work help me understand myself better.” The scale captures two distinct appraisal dimensions. The positive empowerment pathway was measured using the Challenge Appraisal dimension (TCA), comprising 4 items (*α* = 0.87). The negative depletion pathway was measured using the Threat Appraisal dimension (TTA), comprising 5 items (*α* = 0.89).

The dependent variable was older employees’ successful aging at work. Following Mei et al. (68), this study conceptualized successful aging at work as a second-order construct comprising two dimensions: motivation to continue working and capability to continue working. The motivation dimension was measured using the 3-item scale developed by Armstrong-Stassen (69) (*α* = 0.86). The capability dimension was measured using the 4-item scale developed by McGonagle et al. (70) (*α* = 0.86). Participants rated their current work capability across four aspects: overall work ability, physical demands, psychological/mental demands, and interpersonal demands. The two subscales were combined based on theoretical consistency and prior empirical precedent (68). Confirmatory factor analysis confirmed that a two-factor model fit the data well, and the composite reliability of the overall construct was 0.86.

Work autonomy was measured using three items from Breaugh’s (26) nine-item multidimensional scale, specifically the work method autonomy subscale. These three items were: “I am allowed to decide how to go about getting my job done,” “I can choose the methods to use in carrying out my work,” and “I have control over the sequencing of my work activities.” These items were selected because they most directly capture discretion over work methods, which is the aspect of autonomy most relevant to AI-enabled work environments. The Cronbach’s α for this scale in the present study was 0.85.

SOC strategies were measured using the 12-item scale developed by Baltes et al. (29), comprising three dimensions: selection (6 items), optimization (3 items), and compensation (3 items). Following prior research, we used the overall SOC score in the main analyses, computed as the mean of all 12 items. This approach is consistent with the conceptualization of SOC as a general adaptive strategy rather than three independent constructs. The Cronbach’s α for the overall SOC scale was 0.88.

All measures were administered in Chinese. Validated Chinese versions of all scales were used.

Consistent with prior research, gender, age, education, position, and firm type were included as control variables. Gender was coded as 1 = female and 2 = male; age was coded as 1 = 45–55 years and 2 = 56 years and above; education was coded as 1 = high school or below, 2 = college, and 3 = bachelor’s degree or above. Position was treated as an ordered categorical variable reflecting managerial hierarchy (1 = ordinary employee, 2 = junior manager, 3 = middle manager, 4 = senior manager) and entered as a single predictor in the regression models, so that its coefficient captures the increase in outcomes associated with moving up one managerial level. Firm type was a nominal variable and was dummy-coded with state-owned enterprise as the reference category; four dummy indicators were created for private, joint-venture, foreign-owned, and other firms.

To further test the discriminant validity among the core constructs, confirmatory factor analysis (CFA) was conducted using Stata 17.0. The five-factor model demonstrated good fit: χ^2^/df = 1.82, CFI = 0.95, TLI = 0.94, RMSEA = 0.06, SRMR = 0.05. All standardized factor loadings ranged from 0.60 to 0.90 (all *p* < 0.01). The five-factor model significantly outperformed the single-factor model (Δχ^2^ = 1,553, Δdf = 14, *p* < 0.001). Composite reliability (CR) ranged from 0.86 to 0.92, and average variance extracted (AVE) ranged from 0.60 to 0.70, supporting convergent validity. The square roots of AVEs exceeded the inter-construct correlations, supporting discriminant validity.

Given that all variables were collected via a single self-report questionnaire at one time point, common method bias may pose a threat to the validity of the findings. Several procedural remedies were implemented during questionnaire design to mitigate this risk. First, participants were assured of anonymity and confidentiality, and were informed that there were no right or wrong answers. Second, items measuring different constructs were separated and presented in a mixed order rather than grouped by construct. Third, some items were reverse-coded to reduce response consistency. Fourth, different response formats and anchors were used across scales where possible.

Statistically, Harman’s single-factor test was first conducted. The first unrotated factor accounted for 31.78% of the total variance, which is below the recommended threshold of 40%, suggesting that common method bias is not a serious concern. In addition, as reported above, the five-factor measurement model fit the data significantly better than a single-factor model, providing further evidence that the constructs are empirically distinct.

To more rigorously assess common method variance, we conducted an unmeasured latent method factor (ULMC) analysis. The baseline five-factor model showed acceptable fit: CFI = 0.95, TLI = 0.94, RMSEA = 0.06, SRMR = 0.05. The model with the method factor also showed acceptable fit: CFI = 0.96, TLI = 0.95, RMSEA = 0.05, SRMR = 0.04. The addition of the method factor did not substantially improve model fit (ΔCFI = 0.008, ΔTLI = 0.009, ΔRMSEA = -0.004, ΔSRMR = −0.003), as all changes were below the recommended thresholds. These results indicate that common method variance does not pose a serious threat to the validity of the findings.

## 4 Data analysis

### 4.1 Correlation analysis

Pearson correlation analysis revealed that the five core variables—TCA, TTA, work autonomy, SOC strategies, and successful aging at work—were all statistically significantly correlated (see Table 2), providing a foundation for subsequent mediation and moderation effect tests.

**Table 2:** Means, standard deviations, and correlations among key variables (*N* = 558).

Variable	M ± SD	1	2	3	4	5	6	7	8	9
1. Gender	1.52 ± 0.50									
2. Age	1.35 ± 0.48	−0.02								
3. Education	1.62 ± 0.67	0.01	−0.08							
4. Position	1.70 ± 0.94	0.05	−0.04	0.10^*^						
5. TCA (challenge appraisal)	3.62 ± 0.85	0.02	−0.04	0.08	0.14^**^	(0.87)				
6. TTA (threat appraisal)	3.45 ± 0.92	−0.01	0.05	−0.06	−0.10^*^	−0.28^**^	(0.89)			
7. Work autonomy	3.49 ± 0.82	0.06	−0.03	0.09	0.13^*^	0.42^**^	−0.33^**^	(0.85)		
8. SOC strategy	3.58 ± 0.78	0.02	−0.06	0.07	0.11^*^	0.38^**^	−0.25^**^	0.41^**^	(0.88)	
9. Successful aging at work	3.71 ± 0.81	0.12^*^	−0.05	0.07	0.16^**^	0.35^**^	−0.28^**^	0.46^**^	0.43^**^	(0.86)

*p < 0.05, **p < 0.01; diagonal values in parentheses are Cronbach’s α coefficients; M, mean; SD, standard deviation; TCA, Technostress Challenge Appraisal; TTA, Technostress Threat Appraisal; SOC, Selection, Optimization, and Compensation. Control variable coding is reported in the Methods section. Firm type was not included in the correlation matrix because it is a nominal variable and was dummy-coded in the regression analyses; sample distribution of firm type is reported in Table 1.

### 4.2 Baseline regression and path analysis

#### 4.2.1 Main effect tests

Table 3 presents the baseline regression results. After controlling for relevant variables, TCA had a significant positive predictive effect on successful aging at work (*β* = 0.32, *p* < 0.01), supporting H1a. TTA had a significant negative predictive effect (*β* = −0.26, *p* < 0.01), supporting H1b. These results indicate that cognitive appraisal of AI shows a double-edged pattern of association with older employees’ successful aging at work.

**Table 3:** Baseline regression results.

Predictor	Work autonomy			Successful aging at work		
	*β* (SE)	*t*	95% CI	*β* (SE)	*t*	95% CI
Control variables						
Gender	0.08 (0.06)	1.33	[−0.04, 0.20]	0.12 (0.06)	2.00^*^	[0.003, 0.237]
Age	−0.06 (0.07)	−0.86	[−0.20, 0.08]	−0.08 (0.07)	−1.14	[−0.22, 0.06]
Education	0.09 (0.06)	1.50	[−0.03, 0.21]	0.07 (0.06)	1.17	[−0.05, 0.19]
Position	0.10 (0.06)	1.67	[−0.02, 0.22]	0.14 (0.06)	2.33^*^	[0.02, 0.26]
Private enterprise	0.08 (0.06)	1.33	[−0.04, 0.20]	0.11 (0.07)	1.57	[−0.03, 0.25]
Joint venture	0.07 (0.05)	1.40	[−0.03, 0.17]	0.09 (0.05)	1.80	[−0.01, 0.19]
Foreign-owned enterprise	0.06 (0.05)	1.20	[−0.04, 0.16]	0.07 (0.05)	1.40	[−0.03, 0.17]
Others	0.07 (0.05)	1.40	[−0.03, 0.17]	0.10 (0.06)	1.67	[−0.02, 0.22]
Independent variables						
TCA (challenge appraisal)	0.39 (0.07)	5.57^**^	[0.25, 0.53]	0.32 (0.08)	4.00^**^	[0.16, 0.48]
TTA (threat appraisal)	−0.32 (0.08)	−4.00^**^	[−0.48, −0.16]	−0.26 (0.09)	−2.89^**^	[−0.44, −0.08]
Mediator						
Work autonomy				0.45 (0.08)	5.63^**^	[0.29, 0.61]
*R* ^2^	0.31			0.37		
*F*	*F*(10, 547) = 24.60^**^			*F*(11,546) = 29.15^**^		

^*^p < 0.05, ^**^p < 0.01, Firm type was dummy-coded with state-owned enterprise as the reference category.

#### 4.2.2 Mediation effect tests

Bootstrap analyses were conducted with 5,000 resamples using the bias-corrected percentile method. All continuous variables were mean-centered prior to creating interaction terms to reduce multicollinearity. Variance inflation factors (VIFs) for all predictors were below 3.0, indicating no serious multicollinearity concerns.

Mediation analysis using the bootstrap method (Tables 3 and 4) showed that in the resource-gain pathway, the indirect effect of TCA on successful aging at work through work autonomy was 0.18 (SE = 0.05, 95% CI [0.08, 0.28]), supporting H2a. In the demand-loss pathway, the indirect effect of TTA was −0.14 (SE = 0.05, 95% CI [−0.24, −0.04]), supporting H2b.

**Table 4:** Bootstrap mediation effect results.

Pathway	Effect size	SE	95% CI		Significant
			lower	upper	
Resource-gain pathway					
TCA → WA → SAW (indirect effect)	0.18	0.05	0.08	0.28	Significant
TCA → SAW (direct effect)	0.32	0.08	0.16	0.48	Significant
Total effect	0.50	0.08	0.34	0.66	Significant
Demand-loss pathway					
TTA → WA → SAW (indirect effect)	−0.14	0.05	−0.24	−0.04	Significant
TTA → SAW (direct effect)	−0.26	0.09	−0.44	−0.08	Significant
Total effect	−0.40	0.09	−0.58	−0.22	Significant

#### 4.2.3 Moderation and moderated mediation effect tests

Hierarchical regression was used to test the moderating effect of SOC strategies (see Table 5). Results showed that the interaction term TCA × SOC had a significant positive coefficient on work autonomy (*β* = 0.19, *p* < 0.05), supporting H3a. The interaction term TTA × SOC also had a significant positive coefficient (*β* = 0.16, *p* < 0.05). Given that TTA had a negative association with work autonomy, this positive coefficient indicates that SOC strategies buffered its negative impact, supporting H3b.

**Table 5:** Moderating effects of SOC strategy on the relationship between cognitive appraisal of AI and work autonomy.

Predictor	Model 1	Model 2	Model 3
	*β* (SE)	*β* (SE)	*β* (SE)
**Control variables**	Included	Included	Included
TCA (challenge appraisal)	0.39 (0.07)^**^	0.33(0.08)^**^	0.30 (0.07)^**^
TTA (threat appraisal)	−0.32 (0.08)^**^	−0.24 (0.09)^**^	−0.23 (0.08)^**^
SOC strategy (SOC)		0.28 (0.08)^**^	0.26 (0.08)^**^
TCA × SOC			0.19 (0.08)^*^
TTA × SOC			0.16 (0.07)^*^
*R* ^2^	0.31	0.38	0.42
ΔR^2^		0.07	0.04
*F*	*F*(10, 547) = 24.60^**^	*F*(11, 546) = 30.40^**^	*F*(13, 544) = 30.3^**^

^*^p < 0.05, ^**^p < 0.01.

Further Bootstrap tests for moderated mediation (see Table 6) revealed that in the resource-gain pathway, the indirect effect was significant at high SOC levels (effect = 0.22, 95% CI [0.08, 0.36]) but not at low SOC levels (effect = 0.05, 95% CI [−0.05, 0.15]). The index of moderated mediation was 0.09 (95% CI [0.01, 0.17]), supporting H4a. In the demand-loss pathway, the indirect effect was significantly negative at low SOC levels (effect = −0.17, 95% CI [−0.27, −0.07]), but weakened and became non-significant at high SOC levels (effect = −0.03, 95% CI [−0.15, 0.09]). The index of moderated mediation was 0.07 (95% CI [0.01, 0.13]), supporting H4b.

**Table 6:** Moderated mediation effects (Bootstrap analysis).

Moderator level	Indirect effect	SE	95% CI (lower)	95% CI (upper)
Resource-gain pathway: TCA → WA→ SAW				
Low SOC (−1 SD)	0.05	0.05	−0.05	0.15
High SOC (+1 SD)	0.22	0.07	0.08	0.36
Index of moderated mediation	0.09	0.04	0.01	0.17
Demand-loss pathway: TTA →WA→ SAW				
Low SOC (−1 SD)	−0.17	0.05	−0.27	−0.07
High SOC (+1 SD)	−0.03	0.06	−0.15	0.09
Index of moderated mediation	0.07	0.03	0.01	0.13

The indices of moderated mediation reported in Table 6 are based on standardized estimates. The path coefficients reported in Tables 3 and 5 are also standardized. The indirect effects at high and low SOC levels were computed using the bootstrap procedure with 5,000 resamples.

The complete model with all standardized path coefficients is presented in Figures 2, 3. In these figures, direct path coefficients (*β*), indirect effects, and indices of moderated mediation are distinguished by different labels.

**Figure 2:**
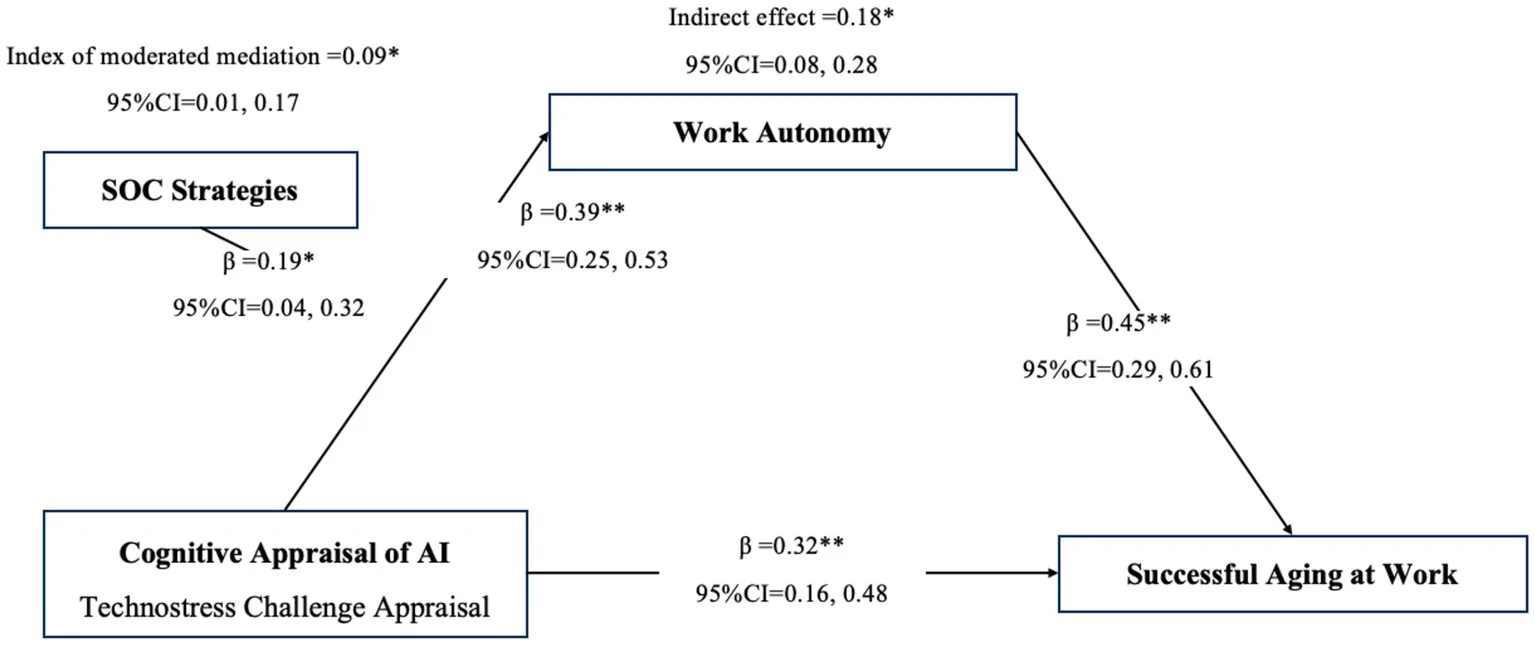
The moderated mediation model for the resource-gain pathway.

**Figure 3:**
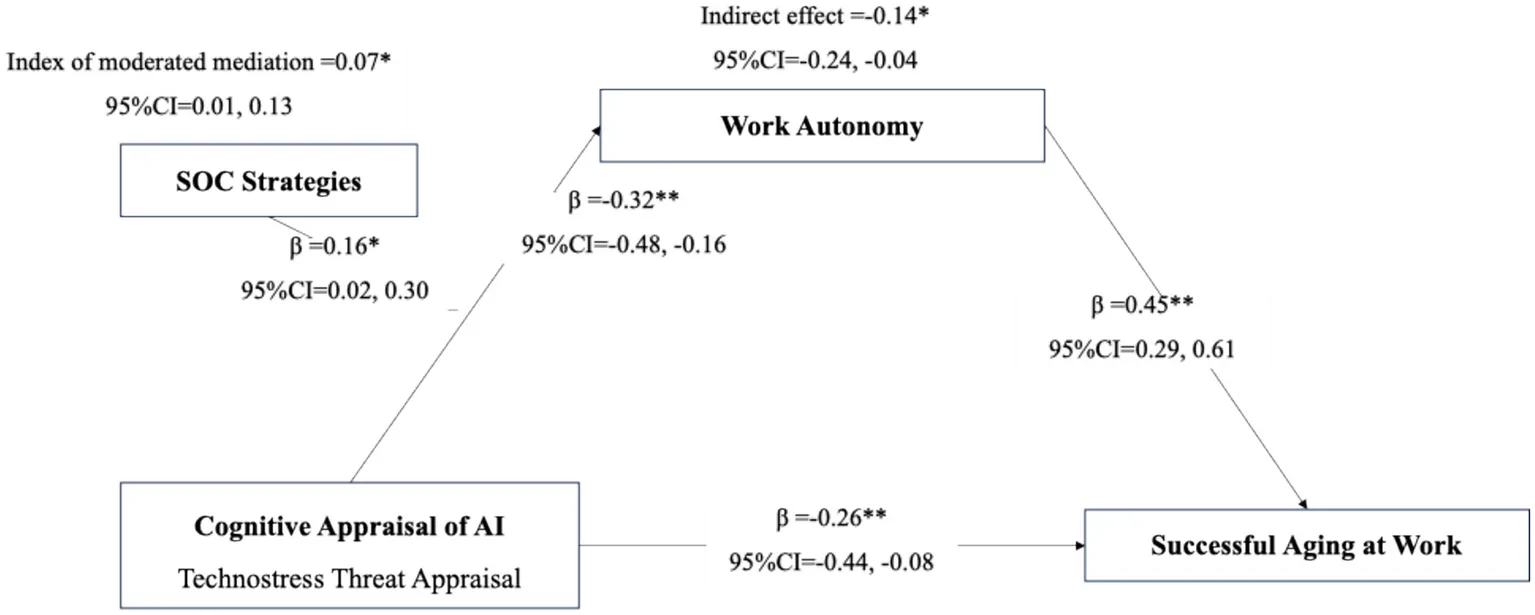
The moderated mediation model for the demand-loss pathway.

To visually illustrate the moderating effects of SOC strategies, we plotted the simple slopes for the relationships between cognitive appraisal of AI (both resource and demand pathways) and work autonomy at high (+1 SD) and low (−1 SD) levels of SOC strategies, following the procedures recommended by Aiken and West (71).

Simple slope tests were conducted to further examine the moderating effects. For the resource-gain pathway, at high SOC levels (+1 SD), the relationship between TCA and work autonomy was significant and positive (simple slope = 0.49, SE = 0.08, *p* < 0.001); at low SOC levels (−1 SD), this relationship was weak and non-significant (simple slope = 0.11, SE = 0.06, *p* > 0.05). The difference between the two slopes was significant (Δ = 0.38, SE = 0.08, *p* < 0.05).

For the demand-loss pathway, at high SOC levels (+1 SD), the negative relationship between TTA and work autonomy was non-significant (simple slope = −0.07, SE = 0.08, *p* > 0.05); at low SOC levels (−1 SD), this relationship was significant and negative (simple slope = −0.39, SE = 0.09, *p* < 0.001). The difference between the two slopes was significant (Δ = 0.32, SE = 0.08, *p* < 0.01).

As shown in Figure 4, when AI was perceived as a job resource (TCA), the positive relationship between TCA and work autonomy was stronger for older employees with high SOC strategies compared to those with low SOC strategies. This pattern indicates that SOC strategies amplify the beneficial effect of AI as a resource on work autonomy, providing visual support for H3a.

**Figure 4:**
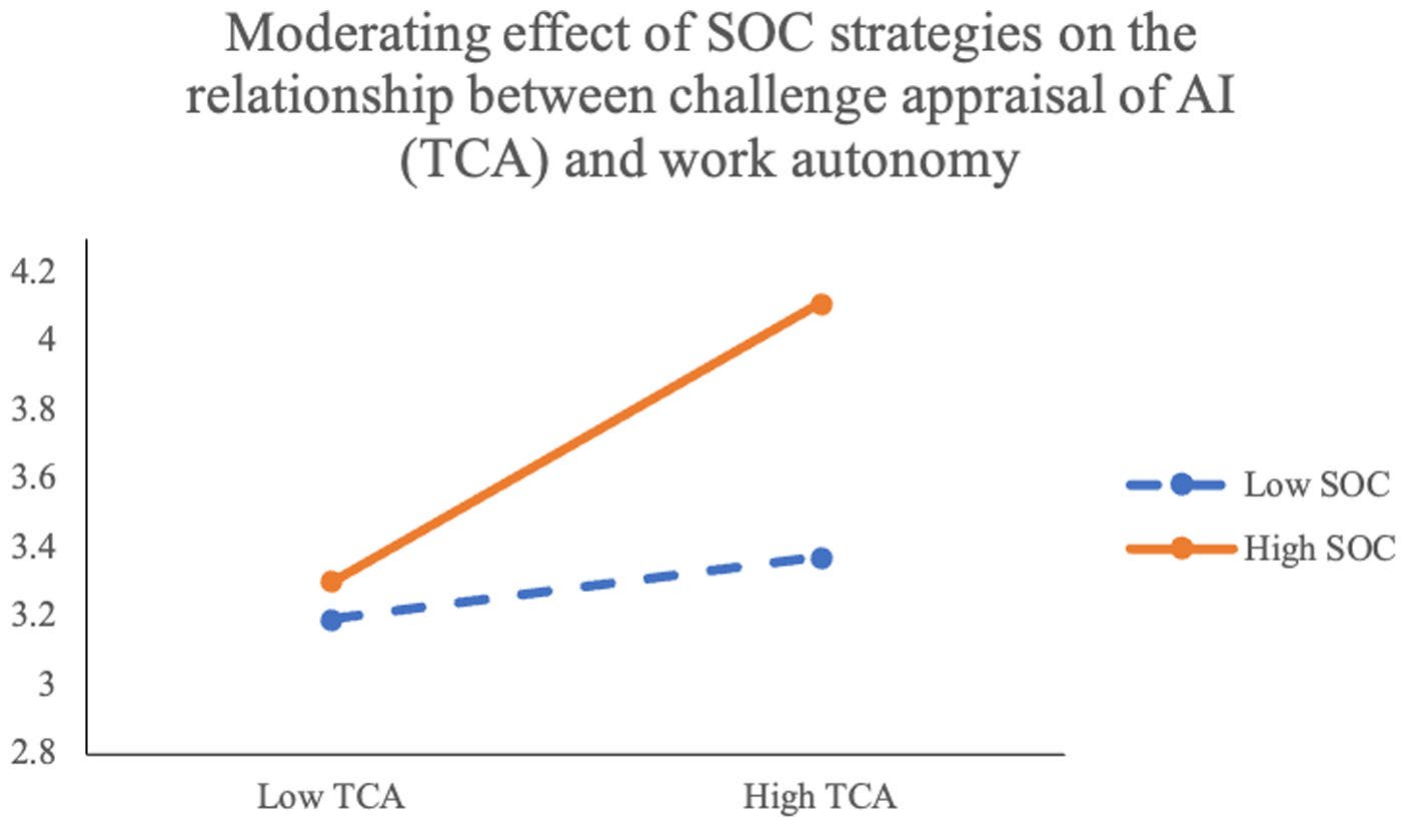
Moderating effect of SOC strategies on the relationship between challenge appraisal of AI (TCA) and work autonomy.

Conversely, as depicted in Figure 5, when AI was perceived as a job demand (TTA), the negative relationship between TTA and work autonomy was significantly attenuated for older employees with high SOC strategies. In contrast, for those with low SOC strategies, the negative slope was steeper, suggesting that SOC strategies effectively buffer the detrimental impact of AI demands on work autonomy. This visual evidence supports H3b.

**Figure 5:**
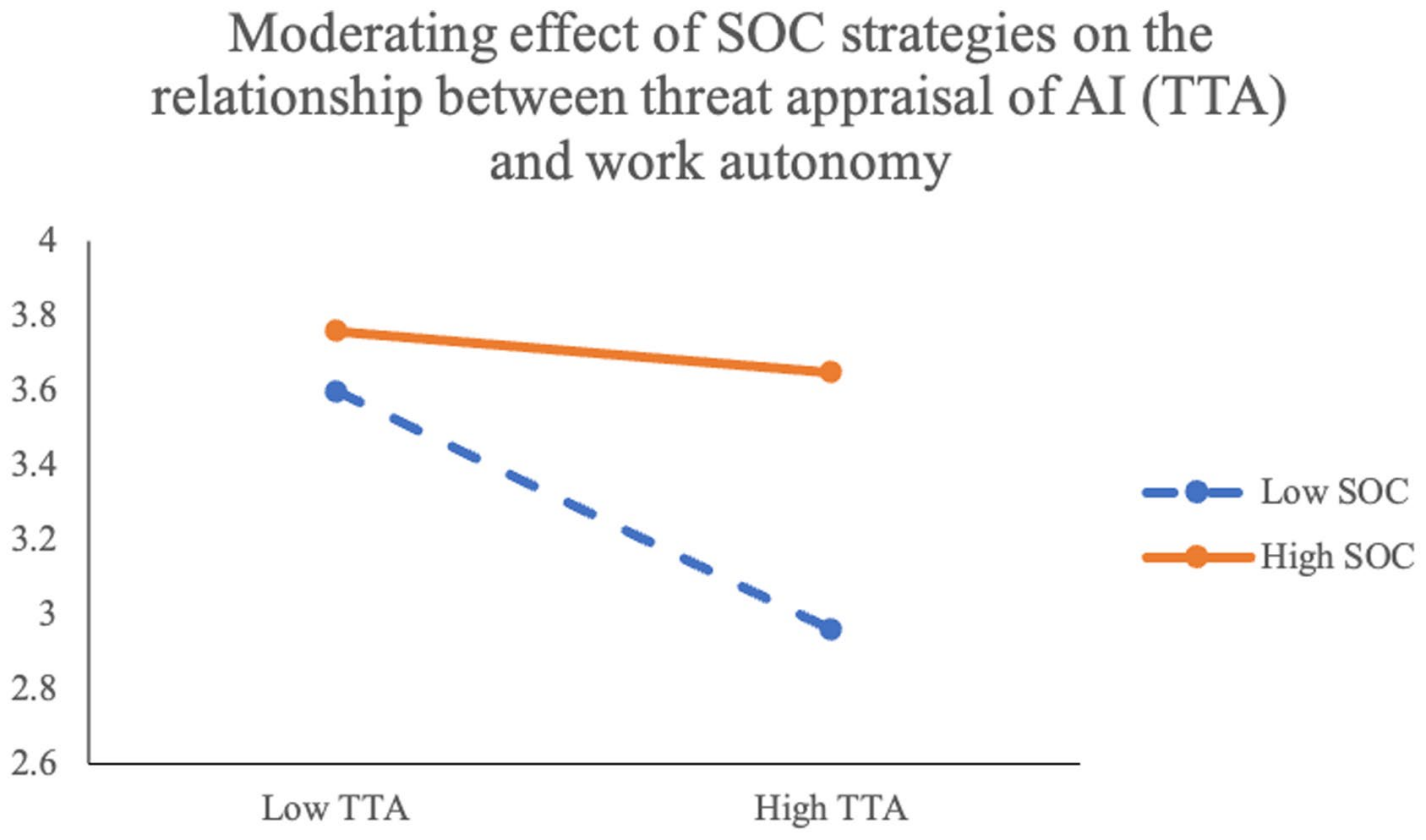
Moderating effect of SOC strategies on the relationship between threat appraisal of AI (TTA) and work autonomy.

#### 4.2.4 Robustness checks

To further verify the reliability of the results, two robustness analyses were conducted (see Table 7). First, structural equation modeling was used to re-estimate all path coefficients, yielding results consistent with the Bootstrap method. For the SEM analysis, we used maximum likelihood estimation. The model included all observed variables and allowed correlations among exogenous variables. Interaction terms were created using mean-centered variables. Model fit was acceptable: χ^2^/df = 1.6, CFI = 0.91, TLI = 0.90, RMSEA = 0.05, SRMR = 0.04.

**Table 7:** Robustness checks: alternative model specifications.

Pathway/Coefficient	Baseline (Bootstrap)	SEM alternative	70% subsample
TCA → WA	0.39 (0.07)^**^	0.37 (0.08)^**^	0.38 (0.09)^**^
TTA → WA	−0.32 (0.08)^**^	−0.29 (0.09)^**^	−0.30 (0.10)^**^
WA → SAW	0.45 (0.08)^**^	0.44 (0.08)^**^	0.45 (0.09)^**^
TCA → SAW (direct)	0.32 (0.08)^**^	0.28 (0.09)^**^	0.29 (0.10)^**^
TTA → SAW (direct)	−0.26 (0.09)^**^	−0.23 (0.09)^**^	−0.24 (0.10)^**^
TCA × SOC → WA	0.19 (0.08)^*^	0.17 (0.08)^*^	0.18 (0.09)^*^
TTA × SOC → WA	0.16 (0.07)^*^	0.14 (0.08)^*^	0.16 (0.09)^*^
TCA → WA → SAW (indirect)	0.18 [0.08, 0.28]	0.16 [0.09, 0.24]	0.17 [0.09, 0.25]
TTA → WA → SAW (indirect)	−0.14 [−0.24, −0.04]	−0.13[−0.21, −0.06]	−0.14 [−0.22, −0.07]

^*^p < 0.05, ^**^p < 0.01.

Second, a random 70% subsample was analyzed. The subsample was drawn using a fixed random seed (seed = 20,260,101) in a single draw. Standard errors are reported alongside path estimates; indirect effects are reported as point estimates with 95% bootstrap confidence intervals. The sign and significance of core variable coefficients remained substantively unchanged, indicating good robustness of the results.

To address the possibility of reverse causality, we tested an alternative model in which successful aging at work predicted AI appraisal through work autonomy. This alternative model fit the data worse than the hypothesized model (Δχ^2^ = 1,562, Δdf = 15, *p* < 0.001; ΔCFI = 0.009, ΔRMSEA = 0.008), providing preliminary support for the hypothesized direction. However, given the cross-sectional design, causal direction cannot be definitively established.

## 5 Discussion

This study employed the JD-R model to analyze the relationship between older employees’ cognitive appraisal of AI and the mediating role of work autonomy and the moderating role of SOC strategies. The findings reveal that older employees’ cognitive appraisal of AI has a dual relationship with successful aging at work, with work autonomy serving as a mediator and SOC strategies as a moderator.

### 5.1 The double-edged pattern of AI appraisal

The study found that the association between older employees’ cognitive appraisal of AI and their successful aging at work depends on whether AI is appraised as a challenge or a threat. Examining both challenge and threat appraisals, we found that appraising AI as a challenge (theoretically corresponding to a job resource) was positively associated with successful aging at work, while appraising AI as a threat (theoretically corresponding to a job demand) was negatively associated with it. This finding resonates with current discussions on the “double-edged sword” effect of AI and is consistent with Conservation of Resources theory, which posits that individuals tend to acquire, maintain, and protect resources; with resource gains driving positive development, and resource losses triggering negative consequences (72).

When AI is appraised as a challenge, its automation and cognitive compensation functions may help older employees mitigate the negative impact of resource depletion. It can replace repetitive tasks, allowing employees to focus on complex and important work (38). Its database function may also serve as a tool for on-demand knowledge verification, opening space for continuous learning and capability advancement. When AI is appraised as a threat, its rapid iteration may create risks of skill obsolescence (43), and the pressure of continuous learning may trigger negative emotions such as anxiety, eroding professional competence (73). Coupled with age-related cognitive changes, older employees may be prone to role confusion (39, 44), ultimately hindering the successful aging process.

### 5.2 The mediating role of work autonomy

Work autonomy mediated both the resource-gain and demand-loss pathways of AI appraisal, with opposite effect directions. This aligns with Self-Determination Theory, which posits that autonomy is a key condition for positive individual development (49).

In the resource-gain pathway, AI appraisal as a challenge may be associated with more flexible work arrangements, helping older employees balance work pace and methods (74). Additionally, their accumulated experience may allow them to autonomously verify AI-generated information, enhancing their sense of control. When older employees have higher autonomy, they may be more willing to learn, making them more inclined to maintain a learning state and proactively acquire new skills to sustain good performance, ultimately achieving successful aging. In the demand-loss pathway, frustrated autonomy may be associated with diminished intrinsic motivation and reduced work engagement. One possible interpretation is that when AI is appraised as a threat, it may be experienced in ways resembling algorithmic management, transforming work into quantifiable metrics and limiting discretionary space (55), ultimately weakening autonomy and hindering successful aging. However, this mechanism was not directly tested in the present study and should be examined in future research.

### 5.3 The moderating role of SOC strategies

This study found that SOC strategies moderate the relationship between cognitive appraisal of AI and work autonomy, functioning as an “amplifier” in the resource-gain pathway and a “buffer” in the demand-loss pathway. This echoes Transactional Theory of Stress and Coping (59) and the JD-R model, suggesting that personal resources can amplify the effects of job resources and buffer the negative impact of job demands.

In the positive pathway, SOC strategies positively moderated the relationship between challenge appraisal of AI and work autonomy. Older employees skilled in using SOC strategies may more easily set task goals and refine operational methods, translating AI assistance into tangible outcomes of heightened work control. In the demand-loss pathway, SOC strategies buffered the negative impact of threat appraisal of AI on work autonomy. Older employees with high SOC levels may allocate resources more efficiently, proactively focus on core tasks, consciously refine work methods, and leverage experience to offset difficulties arising from digital skill deficiencies (30, 75), thereby alleviating the adverse effects of AI pressure on successful aging.

Furthermore, moderated mediation analyses indicated that SOC strategies moderated the mediating role of work autonomy. In the resource-gain pathway, the indirect effect was stronger at high SOC levels; in the demand-loss pathway, the negative indirect effect was mitigated at high SOC levels. This suggests that SOC strategies may serve as an important personal resource that helps older employees coordinate the differentiated effects of AI appraisal on successful aging at work.

## 6 Theoretical and practical contributions of the study

Drawing on the Job Demands-Resources (JD-R) model, this study yields several important theoretical advances and actionable practical implications for organizations in the AI-driven digital workplace.

Theoretically, first, this research enriches existing literature on older employees’ successful aging at work by adopting a cognitive-appraisal perspective to unpack the double-edged correlational pattern of AI. Rather than focusing on objective AI technology itself, we reconceptualize AI as a subjectively appraised workplace stimulus, distinguish challenge appraisal and threat appraisal, and verify their divergent associations with successful aging at work. This extends the application of the JD-R model into digital workplace contexts for aging employees.

Second, this study clarifies the mediating role of work autonomy across both resource-gain and demand-loss pathways. Our findings demonstrate that work autonomy acts as a critical psychological transmission mechanism linking AI cognitive appraisal to successful aging at work, deepening understanding of how job resources function for middle-aged and older staff. Third, we confirm the moderating function of selective optimization with compensation (SOC) strategies. SOC strategies serve as an amplifier in the positive resource-gain pathway and a buffer in the negative demand-loss pathway, further verifying the conditional nature of the moderated-mediation model in the Chinese collectivist cultural setting.

Practically, our results provide targeted human-resource insights for enterprises facing population aging and digital transformation. Organizations should pay attention to older employees’ subjective cognitive appraisal of AI when implementing intelligent workplace systems. Managers are supposed to frame AI as an efficiency-boosting auxiliary tool instead of a substitution threat. Organizational communication and training activities can be designed to help older employees form challenge-oriented perceptions toward AI. Moreover, firms ought to safeguard older employees’ work autonomy and preserve their discretionary space during AI algorithmic management. Finally, enterprises can develop targeted SOC-strategy training for older workers, guiding them to select core work objectives, optimize work procedures, and leverage accumulated experience to compensate for age-related cognitive deficits. Joint efforts from both organizations and individuals can support older employees to maintain sustainable career vitality in the AI era.

## 7 Limitations and recommendations

Despite the theoretical and methodological rigor of this study, several limitations should be acknowledged. The cross-sectional design and the use of self-report data preclude causal inferences. Although we used associational language throughout and tested a reverse causality model, reverse causation cannot be entirely ruled out. For instance, older employees who have already achieved successful aging at work may be more inclined to appraise AI as a challenge rather than a threat. Additionally, all variables were measured via a single self-report questionnaire at one time point, raising concerns about common method variance. Although Harman’s single-factor test and the ULMC analysis indicated that this was not a serious threat, future research should employ longitudinal designs and incorporate multi-source data, such as supervisor ratings or objective performance indicators, to establish temporal ordering and enhance robustness.

This study focused on older employees’ cognitive appraisal of AI rather than objective AI exposure, and several measures were adapted or shortened. Cognitive appraisal and objective exposure are distinct constructs, and this study did not measure the frequency, type, or work embeddedness of AI usage. In addition, age was measured categorically, which may reduce statistical power and obscure finer-grained age effects. Industry category was not recorded, so industry-level heterogeneity could not be examined. Future research should measure objective AI use and subjective appraisal simultaneously, use full and validated scales, collect continuous age data, and adopt stratified sampling across industries.

The generalizability of the findings is limited by the sample and the robustness checks. The sample was drawn exclusively from four cities in Guangdong Province, China, and Chinese culture emphasizes collectivism, which may shape older employees’ attitudes toward AI, their need for work autonomy, and their use of SOC strategies in ways that differ from Western contexts. Future research should conduct cross-cultural comparisons and replicate the theoretical model in different countries and regions to test its external validity. Moreover, the robustness checks, including SEM alternative models and subsample analysis, cannot completely rule out omitted variables or model misspecification. Future research could employ experimental designs or instrumental variable methods to further strengthen causal inferences.

## 8 Conclusion

This study constructs a moderated-mediation framework to examine how older employees’ cognitive appraisal of AI relates to their successful aging at work. Based on the JD-R model, we surveyed 558 employees aged 45 and above from Chinese enterprises. The empirical results reveal that challenge appraisal of AI is positively associated with successful aging at work, whereas threat appraisal is negatively associated with it. Work autonomy mediates both above-mentioned associations. SOC strategies amplify the positive indirect effect in the resource-gain pathway and buffer the harmful indirect effect in the demand-loss pathway.

Our work highlights that older employees’ subjective perceptions of AI, rather than the technology per se, play a critical role in shaping their late-career development outcomes. Organizations should attach importance to employees’ cognition toward artificial intelligence, protect work autonomy, and facilitate adaptive SOC strategies to help aging employees achieve sustainable career development in digital workplaces.

## Data Availability

The raw data supporting the conclusions of this article will be made available by the authors, without undue reservation.
